# PREPAREDNESS NEEDS OF THE FAMILY CARE PARTNER OF THE PERSON WITH DEMENTIA DURING TRANSITION FROM THE HOSPITAL

**DOI:** 10.1093/geroni/igae098.1362

**Published:** 2024-12-31

**Authors:** Marie Boltz

**Affiliations:** The Pennsylvania State University, University Park, Pennsylvania, United States

## Abstract

In the Fam-FFC approach, nurses purposefully engage family care partners in the assessment, decision-making, care delivery and evaluation of function-focused care during hospitalization and the 60-day post-acute period. The goal is to promote functional recovery and well-being in the patient, and prevent unnecessary long-term care admissions, while improving care partners’ preparation for caregiving and well-being. At hospital discharge, on the Preparation for Caregiving Scale, care partners (N=229, 60% female, 59% White, 39% African American) described the least preparation to care for emotional (M= 2.8, SD=1.2) and physical needs (M= 2.9, SD=1.1), deal with the stress of caregiving (M= 2.7, SD=1.2), provide positive activities (M= 2.9, SD=1.1), followed by get the help they need from the health care system (M= 3.11, SD=1.0). Content analysis of nurses’ transitional care notes during the 60 day post-acute period identified five themes that describe care partner needs to support preparedness; they converge with and help explain the quantitative findings. The themes included: psychosocial support for the patient, managing symptoms, physical and cognitive activity, advocacy, and care partner self-care. Findings suggest the need to attend to the psychological and informational needs of care partners, while considering patient well-being and comfort.

